# Avian Influenza H5N1 Screening of Intensive Care Unit Patients with Community-acquired Pneumonia

**DOI:** 10.3201/eid1211.060443

**Published:** 2006-11

**Authors:** Anucha Apisarnthanarak, Pilaipan Puthavathana, Rungrueng Kitphati, Pranee Thavatsupha, Malinee Chittaganpitch, Prasert Auewarakul, Linda M. Mundy

**Affiliations:** * Thammasart University Hospital, Pratumthani, Thailand;; † Siriraj Hospital, Bangkok, Thailand;; ‡ Thai National Institute of Health, Nonthaburi, Thailand;; § Saint Louis University School of Public Health, Saint Louis, Missouri, USA

**Keywords:** avian influenza, H5N1, screening, cost estimate, Thailand, dispatch

## Abstract

From February 1, 2005, to January 31, 2006, we screened 115 adults for avian influenza (H5N1) and influenza A if admitted to an intensive care unit with pneumonia. Using reverse transcription-PCR, viral culture, and serologic testing for anti-H5 antibody, we identified 8 (7%) patients with influenza A (H3N2); none had H5N1. Estimated costs for H5N1 screening were $7,375.

The ongoing avian influenza (H5N1) pandemic poses risks to both human and animal health (*1**–**5*). The potential exists for cross-species transmission of avian influenza to humans and subsequent reassortment of avian and human influenza viruses in coinfected persons (*6*). Although atypical presentations of avian influenza (H5N1) have been reported (*7**,**8*), in most H5N1 case-patients pneumonia was the primary condition (*3**,**4*). To assess the prevalence of avian influenza (H5N1) and influenza A pneumonia, we screened adults admitted to a medical intensive care unit (ICU) with community-acquired pneumonia (CAP) for H5N1 and calculated the cost estimates for H5N1 screening in a tertiary care center of an H5N1-endemic area in Thailand.

## The Study

Thammasat University Hospital is a 450-bed tertiary care center with an 8-bed intensive care unit (ICU) equipped with central air-conditioning and 2 isolation rooms. The hospital serves a 150-km radius referral base in central Thailand and has 980 healthcare workers (HCWs). Annual influenza vaccination was not routinely offered to HCWs. During the study period, 2 confirmed cases of H5N1 occurred within 150 km of our hospital.

All adults admitted to the ICU with CAP between February 1, 2005, and January 31, 2006, were eligible for enrollment. Tracheal aspirates were collected for H5N1 testing, with reverse transcription (RT)-PCR, and viral culture. In patients <60 years with >14 days survival posthospitalization, paired acute-phase and convalescent-phase serum specimens were collected for identifying anti-H5 antibody. Acute-phase serum specimens for determining anti-H5 antibody were obtained within 1 week of symptoms, while convalescent-phase serum specimens were obtained >14 days after the acute-phase specimens were collected. Data collection included demographic characteristics, clinical data, and the costs associated with H5N1 screening. The diagnosis of CAP was defined according to the criteria recommended by the American Thoracic Society (*9*). Patients who were hospitalized for >2 days and in whom pneumonia developed were excluded from this study. The current Thai national surveillance definition for probable avian influenza (H5N1) included the following: 1) presence of fever (>38°C), and 2) influenza-like illness, and 3) exposure to sick poultry or residence in the disease-endemic areas with excess poultry death rates, and 4) radiographic evidence of severe CAP without an identified etiologic agent (*10*).

Viral cultures for H5N1 and influenza A, as part of screening, were incubated in Madin-Darby canine kidney (MDCK) cell monolayers at the Thai National Institute of Health. Tracheal aspirate specimens were tested by an RT-PCR assay specific for the hemagglutinin gene of H5 (*11*). If a specimen yielded a positive H5 band, the specimens were confirmed by different RT-PCR primers and by real-time RT-PCR (*12*). All serum samples were tested for H5-specific antibody by a microneutralization (micro-NT) test. The reactive samples underwent confirmatory immunofluorescence testing by using H5-transfected 293 T cells as the test antigen (*13*). Influenza A/Thailand/1(KAN-1)/2004 (H5N1) was used as the test virus. Acute-phase and convalescent-phase serum samples were serially diluted from 1:20 to 1:80. On the basis of previously established criteria, a positive test was defined as a neutralizing antibody titer >80 with a confirmatory immunofluorescence assay (*14*). Adults >60 years of age were excluded from the serologic tests because the H5N1 micro-NT was previously reported to be less specific in this population (*14*).

Laboratory diagnostic costs (RT-PCR for H5N1, viral culture, and paired acute- and convalescent-phase serum samples for anti-H5 antibody) for each patient were obtained from line-item reports of the hospital's fiscal system. All costs in Thai baht currency were converted to US dollars at an exchange rate of 40 bahts per 1 US dollar. The cost for isolation of the index case, if influenza A or avian influenza (H5N1) was detected, were calculated from prior cost estimates (*15*).

One hundred fifteen of 450 patients (25%) met the definition of CAP and consented to study participation. The patient characteristics are summarized in Table 1. None of the 115 patients had tracheal aspirates positive for H5N1; also not positive were any serologic test results from the 42 patients (37%) who were <60 years old and survived >14 days after hospitalization. We were unable to calculate the prevalence of anti-H5 antibody in this sample, given that only 37% of participants underwent complete diagnostic antibody testing.

**Table 1:** Demographic and clinical data for 115 hospitalized adults with severe community-acquired pneumonia at a tertiary care center in an H5N1-endemic region of Thailand*

Characteristics	Total (N = 115)		Influenza A H3N2 (n = 8)	Without concomitant influenza A H3N2 (n = 107)	p value†
Age, years (mean, range)	64 (17–82)		72 (55–82)	64 (17–74)	0.06
Sex, male	48 (42)		4 (50)	44 (41)	NS
Tobacco smoking	21 (18)		1 (12)	20 (19)	NS
No. of comorbid conditions (median, range)	1 (0–4)		3 (1–4)	1 (0–3)	<0.001
Underlying diseases‡§					
Lung disease	48 (42)		4 (50)	44 (41)	NS
Diabetes	25 (22)		2 (25)	23 (21)	NS
Cardiovascular	14 (12)		1 (12)	13 (12)	NS
Cerebrovascular or other neurologic disease	12 (10)		1 (12)	11 (10)	NS
Other	42 (37)		3 (38)	39 (36)	NS
Initial clinical symptoms§					
Pulmonary¶		108 (94)	7 (87)	101 (94)	NS
Gastrointestinal#		8 (7)	4 (50)	4 (4)	0.001
Neurologic**		9 (8)	1 (12)	8 (8)	NS
Other		2 (2)	0 (0)	2 (1)	NS
APACHE-II score, median (range)		16 (9–22)	17 (9–22)	15 (9–22)	NS
History of recent travel		0	0	0	NA
Met definition of probable H5N1		18 (16)	2 (25)	16 (15)	NS
History of exposure to index case		0	0	0	NA
Outcome					
Death††	12 (10)		7 (88)	5 (5)	<0.001
LOS in MICU	14 (1–46)		15 (1–46)	14 (2–42)	NS
H5N1 seroconversion	0		0	0	NA

*Data are no. (%) of patients, unless otherwise indicated; NS, nonsignificant; NA, nonapplicable; APACHE-II score, Acute Physiology and Chronic Health Evaluation Score II; LOS, length of stay; MICU, medical intensive care unit. †Categorical variables were compared using χ^2^ or Fisher exact test, as appropriate; Continuous variables were compared using the Wilcoxon rank sum test or *t* test, as appropriate. All p values were 2-tailed; p<0.05 was considered significant. ‡Included those considered by the Advisory Committee on Immunization Practices of the Centers for Disease Control and Prevention to be associated with an increased risk of complication from influenza infection. §Most patients had multiple underlying diseases and initial clinical symptoms so the sums of all percentages are >100%. ¶Included cough, dyspnea or tachypnea, rigor and/or chills, pleuritic chest pain, purulent sputum, or changes in the characteristics of sputum, and auscultatory findings. #Included diarrhea, and/or nausea or vomiting, abdominal tenderness. **Included drowsiness, confusion, coma. ††All patients did not receive antiviral therapy.

Eighteen patients (16%) met the Thai national surveillance definition of probable H5N1, yet tracheal aspirates and serologic test results were negative for H5N1. The median time from initial symptoms to hospitalization was 4 days (range 2–8 days), and all 18 were appropriately placed on contact and droplet isolation; the mean duration of isolation was 9 days (range 4–13 days).

Although 48 (42%) of the 115 participants had no identified etiologic agent associated with CAP, Streptococcus pneumoniae (n = 39; 34%), influenza A (H3N2) (n = 8; 7%), Staphylococcus aureus (n = 7; 6%), and Haemophilus influenzae (n = 6; 5%) were the most common microorganisms detected. In addition, 19 patients (n = 19; 16%) had gram-negative microorganisms detected. All patients with H3N2 pneumonia were promptly transferred to an isolation room; 5 (62.5%) had dual infections of H3N2 and S. aureus (n = 3), Klebsiella pneumoniae (n = 1) and Pseudomonas species (n = 1), while CAP due to H3N2 developed in 3 (37.5%). Of 18 patients who met the definition of probable H5N1, 8 (44.5%) had S. pneumoniae infection, 4 (22.5%) had S. aureus infection, 2 (11%) had H3N2 infection, 2 (11%) had Burkholderia pseudomallei infection, and 2 (11%) had no other agent detected. No CAP patients had anti-H5 antibody seroconversion, although 1 participant had evidence of positive anti-H5 antibody with low titer (*10*) during the recovery phase. This patient lived in an avian influenza (H5N1)–endemic area without a documented excess poultry death rate, and reported no exposure to sick poultry or persons with suspected avian influenza (H5N1) infection. His tracheal culture yielded S. pneumoniae. All patients with H3N2 pneumonia sought treatment between late March and November, the influenza A season in Thailand, and were in contact and droplet isolation for a mean of 7 days (range 1–12 days). The all-cause mortality rate was 10% (Table 1). The cost estimates were $7,375 for H5N1 screening, $23,328 for subsequent infection control measures, $300 for annual influenza vaccination of ICU HCWs, and $9,800 for annual influenza vaccination of the entire hospital staff (Table 2). The perceived benefits of vaccination of all ICU HCWs included reduced risk for influenza among vaccinated HCWs and reduced risk for influenza transmission to at-risk ICU patients.

**Table 2:** Cost estimates for routine avian influenza (H5N1) surveillance, laboratory diagnostics, and infection control measures in the ICU, February 1, 2005 – January 31, 2006*

Category	No. measures	Estimated cost (US$)	Total (US$)
Cost associated with H5N1 routine screening			
Diagnostic testing			
RT-PCR	115	$25 × 115	2,875
Viral culture	115	$30 × 115	3,450
Paired acute- and convalescent-phase serology for anti-H5 antibody†	42	$25 × 42	1,050
Isolation for probable H5N1 (n = 18)‡			
Gowns/d	1,800	$1/gown × 1,800 × 9 d	16,200
Gloves/d	1,800	$0.05/pair × 1,800 × 9 d	810
Surgical masks/d	1,800	$0.25/mask × 1,800 × 9 d	4,050
Staff time (min/d) to put on/take off gloves, gowns, and mask	1,800	$1.26/hour × 1,800	2,268
Cost of universal influenza vaccination			
ICU HCWs	30	$10 × 30	300
HCWs, entire hospital	980	$10 × 980	9,800

*ICU, intensive care unit; RT-PCR, reverse transcriptase polymerase chain reaction; HCWs, healthcare workers. †All 115 patients had acute phase serum samples tested for anti-H5 antibody; 42 (37%) were <60 years old and survived >14 days after hospitalization. ‡Estimated 1 min to put on and take off the protection gear with 100 encounters per day (*15*).

## Conclusions

Our study findings are relevant to the prevention and control of spread of both H5N1 and H3N2. The relatively high prevalence of H3N2 (7%) among our CAP patients suggests that HCWs in ICUs in disease-endemic regions are at high-risk of acquiring influenza A. An annual influenza vaccination occupational health program, similar to those in developed countries, along with targeted case identification of patients at high risk for influenza pneumonia, may help minimize the clinical and economic consequences of influenza A transmission. Although the importance of a single patient's positive low-titer anti-H5 antibody in this study was uncertain, this finding may represent a false-positive test, given that the patient had no notable exposure to sick poultry or to persons with suspected H5N1 infection. Additionally, the fact that all 18 probable case-patients had negative results for H5N1 suggests that the current Thai surveillance definition may need further refinement. Given the potential for reassortment of H5N1 and influenza A in a coinfected person residing in a disease-endemic setting, additional H5N1 screening, along with cost-effectiveness studies, are warranted before this screening strategy is adapted to H5N1-endemic areas.
